# Screening Atg11, a central organizer of yeast selective autophagy, for residues involved in the Atg11-Atg9 interaction

**DOI:** 10.17912/micropub.biology.001941

**Published:** 2026-05-29

**Authors:** Chimi Dolker Sherpa, Patricia Woghiren, Erin Leonello, Mina Abdel-Khalek, Benjamin J. Schuessler, Josh V. Vermaas, Steven K. Backues

**Affiliations:** 1 Chemistry, Eastern Michigan University; 2 MSU-DOE Plant Research Laboratory, Michigan State University

## Abstract

The yeast protein Atg11, whose structure is not fully solved, is a central organizer of autophagosome formation that recruits Atg9 during selective autophagy. Although the residues in Atg9 responsible for this interaction are known, those in Atg11 are not. In an attempt to discover the binding site of Atg9 on Atg11, we screened a number of mutants within amino acid residues 455-627 of Atg11, guided in part by an AlphaFold2-generated model of the Atg11 dimer. However, we were not able to identify specific residues essential for the interaction with Atg9, suggesting that the binding region may lie elsewhere on Atg11.

**Figure 1. Mutations in the 455-535 and 576-627 regions of Atg11 do not cause full loss of interaction with Atg9:** **(A)** Schematic representation of Atg11 showing the coiled-coil domains (black boxes) and the CLAW domain (grey box), with a.a. shown by italicized numbers. Fragments previously reported to bind or not bind with Atg9 are indicated. **(B)** Yeast 2 hybrid assay of BD-Atg9 with the indicated AD-Atg11 mutants. Non-selective media is SMD -ura -leu; selective media is SMD -ura -leu -his +1.5 mM 3-AT. On selective media, (+) indicates growth and (-) indicates no growth across multiple replicates of the assay. AD-EV = pGAD empty vector; other mutants as described in text. **(C)** Alignment analysis of the conservation of Atg11 residues 467-469 across yeast species where the Atg11-interacting residues of Atg9 are conserved or are not as conserved. **(D)** Front and side view of the flask-shaped Atg11 dimer and **(E)** front view of the Z-shaped Atg11 dimer predicted with AlphaFold2 Multimer. **(F)** pLDDT confidence map of the flask-shaped Atg11 structure. **(G)** Close-up of flask-shaped dimer showing predicted locations of YDLL residues. **(H)** Surface rendering of flask-shaped model highlighting predicted surface exposed residues in the 455-535 region. **(I)** CoIP of various overexpressed GFP-Atg11 mutants by overexpressed Atg9-PA. Blots were probed with anti-GFP, which also detects the PA tag. **(J)** Quantitative analysis of CoIP results. The Atg11 to Atg9 band intensity ratio was normalized to the values from WT for each replicate. For the CoIP graph, the GFP-Atg11 band intensity of the 𝚫CC2 samples, which are known to show no binding (Meyer et al., 2022), were used as the background and subtracted from the GFP-Atg11 band intensity of the other samples before normalizing. Error bars are 95% confidence intervals.

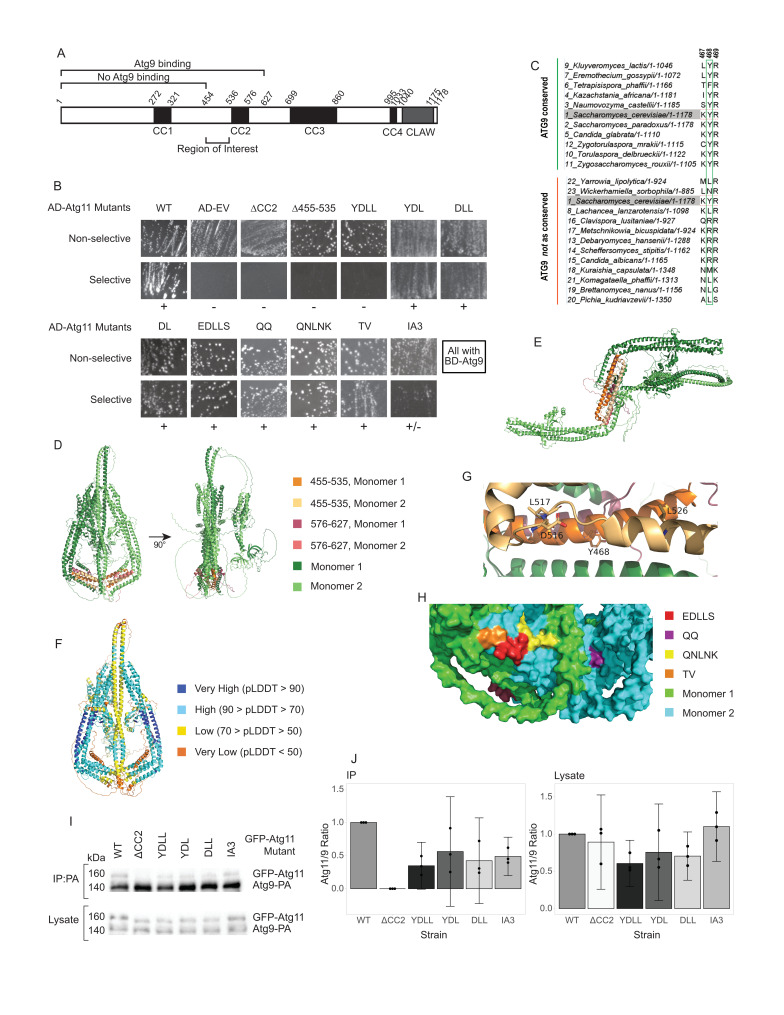



## Description

Macroautophagy (hereafter referred to simply as autophagy) is a critical stress-response pathway conserved in all eukaryotes. Bulk autophagy, triggered by starvation, semi-randomly degrades materials from the cytoplasm in order to reuse the nutrients for cellular survival. In contrast, selective autophagy is not primarily regulated by a need for nutrients but targets specific cytoplasmic cargo to the lysosome (in animals) or vacuole (in other eukaryotes) for degradation (Xie & Klionsky, 2007). Selective autophagy contributes to the health of the cell by breaking down intracellular pathogens, protein aggregates, and malfunctioning organelles. In humans, it is particularly important for long-lived cells such as neurons and helps prevent neurodegenerative diseases such as Parkinson’s and Alzheimer’s (Choi et al., 2013; Lu et al., 2022). For this reason, there is great interest in understanding the proteins that control selective autophagy and how they interact. Much of this research has focused on autophagy in the model eukaryote *Saccharomyces cerevisiae,* baker’s yeast.

Selective autophagy in yeast begins when cargo receptor proteins are recruited to a specific autophagic cargo. These receptors bind to Atg11, the central organizer of the autophagy initiation complex during selective autophagy (Yorimitsu & Klionsky, 2005; Zientara-Rytter & Subramani, 2020). This then arranges the proteins that start the process of building the autophagosome - the double-membrane vesicle that will envelop the cargo and deliver it to the vacuole for degradation.

The structure of Atg11 is not fully solved, nor is the manner by which it recruits and organizes the autophagic machinery fully understood. It is predicted to be a dimer with many coiled-coil domains (Yorimitsu & Klionsky, 2005). Biophysical data has verified it to be a rod-shaped parallel dimer (Suzuki & Noda, 2018), and a crystal structure of a portion of coiled-coil domain 3 shows this to be a flexible coil (Margolis et al., 2020). Atg11 binds to cargo receptors via its C-terminal claw domain, the structure of which has been solved for its human homolog FIP200 (Turco et al., 2019). A cryo-EM structure of the N-terminal half of the FIP200 protein is also available (Chen et al., 2025); however, the N-terminal region of FIP200 is not homologous to that of Atg11. This region binds to core autophagic machinery such as the kinase Atg1 and the scramblase Atg9 (He et al., 2006; Meyer et al., 2022; Yorimitsu & Klionsky, 2005). Atg9 is a trimeric transmembrane protein that is found in populations of small vesicles, and Atg11 binding to Atg9 recruits these vesicles and causes their fusion to form the phagophore (Backues et al., 2015; Yamamoto et al., 2012). Atg9 equilibrates lipids delivered from the ER to both bilayers of the phagophore membrane; as such, it is critical for the formation of the autophagosome (Guardia et al., 2020; Orii et al., 2021; Sawa-Makarska et al., 2020). 

Atg9 has been shown to interact with Atg11 via two PLF (Pro-Leu-Phe) motifs in its N-terminal unstructured region: PLF ^163-165^ and PLF ^187-189^ (Coudevylle et al., 2022). A neighboring histidine, H192, is also involved (He et al., 2006), and mutation of these residues blocks selective autophagy by preventing recruitment of Atg9 to the site of autophagosome formation (Coudevylle et al., 2022; He et al., 2006). However, it is unknown which residues in Atg11 interact with Atg9.

Previous studies have shown that amino acid residues (a.a.) 1-627 of Atg11 are sufficient for binding to Atg9 (He et al., 2006) while a.a. 1-454 are not (Hollenstein et al., 2021), suggesting that the Atg9 binding site might be between a.a. 455-627. Our lab previously investigated part of this region: a.a. 536-576, termed coiled-coil domain 2 (CC2), which had initially been implicated as a likely site for the binding of both Atg1 and Atg9 as well as Atg11 dimerization (Yorimitsu & Klionsky, 2005). However, our systematic mutagenesis data suggested that CC2 is more likely to be involved in overall folding and stability of the N-terminus of Atg11 (Meyer et al., 2022). Therefore, we focused on a.a. 455-535, as that region had not been previously investigated ( Figure 1A ).

Yeast-2-Hybrid (Y2H) results showed that atg11Δ455-535 lost interaction with Atg9, just like atg11ΔCC2 ( Figure 1B ). We then examined the sequence of the 455-535 region across various yeast species, looking for residues that are conserved in the family *Saccharomycetacea* , where the Atg11-binding region of Atg9 is conserved, but not conserved in other species from order *Saccharomycetales* where the Atg11-binding region of Atg9 is not conserved ( Figure 1C ). This analysis yielded four residues: Y468, D516, L517 and L526. A quadruple mutant of all of those residues to alanine (YDLL) mostly lost interaction with Atg9, though a little bit of growth could still be detected in the Y2H assay. However, subsets of 2 or 3 of these mutations (YDL, DLL, or DL) did not lose interaction in this assay, suggesting that none of these was a critical residue ( Figure 1B ).

We used AlphaFold2 Multimer to predict the structure of the Atg11 dimer. The top-ranked model was a “flask-shaped” parallel dimer ( Figure 1D ). However, some other models predicted a Z-shaped dimer ( Figure 1E ). Both models showed extensive monomer-monomer interactions in CC2, CC3 and CC4, consistent with previous reports that both the N- and C- termini of Atg11 could dimerize (Suzuki & Noda, 2018). They both suggested that CC1 and CC2 formed an L-shaped helical bundle, with CC3 and the C-terminus connected by disordered linkers; however, the placement of CC3 and the C-terminus varied between these models - in the flask-shaped, they were neatly packed against each other, while in the Z-shaped dimer, they were placed at random angles, not interacting with CC1, CC2, or each other. The pLDDT for both models was relatively low, particularly at a critical interface at the bottom of the flask-shape, raising the possibility that Atg11 can take on multiple conformations ( Figure 1F ).

Y468 and L526 were placed on the interior of the Atg11 dimer in both AlphaFold models, which would not allow a direct role in Atg9 binding, while D516 and L517 were partially surface exposed ( Figure 1G ). We mutated residues E515-S519 to alanine (EDLLS) to target that entire surface, but this mutant did not lose interaction with Atg9 ( Figure 1B ). We also generated three additional mutants, QQ (Q475A, Q476A), QNLNK (Q493A, N494A, L496A, N500A, K501A), and TV (T511A, V512A), all of which targeted sets of residues within the 455-535 region that the AlphaFold models predicted to be surface exposed ( Figure 1H ). However, none of these mutants lost interaction, either ( Figure 1B ).

The region downstream of CC2 was primarily disordered in our AlphaFold models. To determine if this region was important for Atg9 binding, we replaced residues 576-627 of Atg11 with residues 2-53 of an intrinsically unstructured protein, PAI3. The Atg11_IA3 mutant showed a partial loss of interaction with Atg9 by yeast-2-hybrid ( Figure 1B ).

Finally, we confirmed our yeast-2-hybrid results by performing co-immunprecipitation (CoIP) assays between Atg9-PA and selected GFP-Atg11 mutants. These were performed in growing conditions, where this interaction has previously been assessed (Coudevylle et al., 2022; He et al., 2006; Meyer et al., 2022). These showed a partial loss of interaction between Atg9 and all of the Atg11 mutants tested - YDLL, YDL, DLL and IA3. In addition, there was a reduction of the levels of the YDLL, YDL and DLL mutants in the lysates, suggesting that these mutations might be detrimental to the overall stability of Atg11 ( Figure 1I, J).

Overall, none of the residues or regions we mutated emerged as strong candidates for an Atg9 binding site. The YDLL mutant showed a loss of interaction only when all four residues were mutated, and CoIP results showed that this was only a partial loss of interaction. The fact that these residues are predicted to be in different locations on the folded protein and two of them are predicted to be interior-facing, along with the lower expression observed for this mutant, suggests that they have a primary effect on Atg11 folding and only a secondary effect on Atg9 binding. Similarly, the fact that replacement of 52 residues with those of an unrelated protein led to only a partial loss in binding suggests that the a.a. 576-627 is also not the binding site for Atg9. Interestingly, a recent study showed that an overlapping region, a.a. 612-646, has membrane-binding properties, supporting a different role for this part of Atg11 (Andhare et al., 2025).

We have not exhaustively mutated a.a. 455-627, so we can not rule out the possibility that Atg9 binds in this region. However, we have mutated the most likely candidate residues without finding any specifically responsible for Atg9 binding, suggesting that the core Atg9 binding region may lie elsewhere in the Atg11 N-terminus, while a.a. 455-627 have an indirect effect. The AlphaFold multimer model predicts a.a. 454-591 to be a dimerization interface for Atg11; therefore, we speculate that the removal of a.a. 455-627 from the N-terminal construct may disrupt its proper folding and dimerization, leading indirectly to a loss of Atg9 binding.

## Methods

***Sequence analysis to identify candidate Atg9-binding residues:*** Atg9 homologues in 23 yeast species were scored as “conserved” or “not conserved” based on the conservation of residues known to be involved in binding of Atg11 (H ^192^ , PLF ^163-165^ , and PLF ^187-189^ ). Then, an alignment of Atg11 residues 455-535 in the same species was manually screened for residues that were conserved only in species where Atg9 was conserved. A full description of this process and the alignments can be found in the supporting data at https://osf.io/rnx7v .

***Strain and plasmid construction:*** The pGAD-ATG11 mutant plasmids were ordered from GenScript. The pRS416-Cu-GFP-ATG11 mutant plasmids (YDLL, YDL, and DLL) were made using InFusion cloning. The vector pRS416-Cu-GFP-ATG11 was digested with EcoRI-HF (NEB), which cuts at two endogenous EcoRI sites in Atg11. The insert was amplified by PCR using Phusion polymerase (NEB) from the respective pGAD-ATG11 mutant with primers 5’-AGAAGAAGAAGAATTCAATAGTCAAG-3’ and 5’-ATTTGCTATAGAATTCTCATTTTCATC-3’, ordered from IDT. The vector and insert were combined by InFusion reaction (Clontech, Takara Bio USA Inc.) and transformed into E. coli DH5ɑ (Stellar Competent Cells - HST08 strain) and plated on LB carbenicillin plates for colony growth. pRS416-Cu-GFP-ATG11 ^IA3^ was generated by similar methods, except the digest was performed with AvrII and HindIII and the primers used were 5’-CAGATGGAAATCCTAGGTATTA-3’ and 5’-CAACTTCATAAAGCTTATTGTC-3’.

Plasmids were purified using Purelink plasmid miniprep kits (ThermoFisher), verified by Sanger sequencing (Eton Biosciences) and/or whole plasmid sequencing (Plasmidsaurus) and introduced into yeast cells using standard transformation procedures (Gietz & Schiestl, 2007).

***Yeast-2-hybrid:*** Yeast multiple-knockout cells (lacking 25 *ATG* genes to minimize indirect or interfering interactions; YCY149) expressing BD-ATG9 and AD-ATG11 or mutants thereof were patched on both selective (SMD -ura -leu -his w/ 1.5 mM 3-amino-triazol) and nonselective (SMD -ura -leu) plates and grown for 4–5 days at 30°C before imaging. Results shown are representative of at least five replicates per strain (except for AD-EV and QNLNK, where only two replicates were performed). Full results of all replicates and representative images of complete plates can be found in the supporting data at https://osf.io/4h6bz/ .

***Co-Immunoprecipitation and Western Blotting:*** Yeast were grown under nutrient rich conditions using SMD -ura -trp media at 30°C with shaking at 300 rpm, and harvested while in log phase (OD _600_ < 1). Co-immunoprecipitation was carried out exactly as described previously (Meyer et al., 2022) except that all washes were performed in the 4℃ cold room to help prevent breakdown. Precast 4-15% gradient SDS-PAGE gels (Bio-Rad) and BLUEstain2 protein ladder (GoldBio) were used. The electrophoresis was run for ~3 hours, starting at 80 V and increasing to 120 V after initial band separation. Gels were transferred to PVDF membranes via tank transfer, which was run for 1 hour at 120 V. Membranes were incubated in blocking solution (4% non-fat dry milk in tris-buffered saline). To detect Atg9-PA tag pulldowns, 1:500 mouse anti-GFP primary antibody (Takara; JL-8) and 1:5000 peroxidase-conjugated goat anti-mouse secondary antibody (Jackson ImmunoResearch; 115-035-003) were used.

***Structural Modeling:*** To develop structure-based hypotheses for potential interaction sites between Atg9 and Atg11, AlphaFold-Multimer version 2.3.2 (Evans et al., 2022) was used to generate 25 potential interaction complexes. These complexes were optimized using the FoldX4 “repair” function (Delgado et al., 2019). Visualization was performed in PyMol 3 (Schrödinger, LLC, 2015).

## Reagents

**Table:** 

***Saccharomyces cerevisiae* strains used in this study **		
**Name**	**Genotype**	**Source**
atg11Δ	*MATα* *his3Δ200 leu2-3,112 lys2-801 suc2-Δ9 trp1Δ901 ura3-52 atg11Δ::KanMX*	Meyer et al., 2022
YCY149	*MATα* *his3-Δ200 leu2-3,112 lys2-801 trp1-Δ901 suc2-Δ9 ura3-52 atg1Δ, 2Δ, 3Δ, 4Δ, 5Δ, 6Δ, 7Δ, 8Δ, 9Δ, 10Δ, 11Δ, 12Δ, 13Δ, 14Δ, 16Δ, 17Δ, 18Δ, 19Δ, 20Δ, 21Δ, 23Δ, 24Δ, 27Δ, 29Δ gal4Δ gal80Δ GAL1-HIS3 GAL2-ADE2 met2∷GAL7-lacZ atg31Δ∷KanMX*	Cao et al., 2009
**Plasmids used in this study**		
**Name**	**Description**	**Source**
pGBDU-Atg9	BD vector with full length Atg9	He et al., 2006
pGAD-ATG11	AD vector with full length WT Atg11	Meyer et al., 2022
pGAD-ATG11ΔCC2	AD vector with atg11∆ ^536-576^	Meyer et al., 2022
pGAD-ATG11Δ455-535	AD vector with atg11∆ ^455-535^	This study
pGAD-ATG11 ^YDLL^	AD vector with atg11 ^Y468A, D516A, L517A, L526A^	This study
pGAD-ATG11 ^YDL^	AD vector with atg11 ^Y468A, D516A, L517A^	This study
pGAD-ATG11 ^DLL^	AD vector with atg11 ^D516A, L517A, L526A^	This study
pGAD-ATG11 ^DL^	AD vector with atg11 ^D516A, L517A^	This study
pGAD-ATG11 ^EDLLS^	AD vector with atg11 ^E515A, D516A, L517A, L518A, S519A^	This study
pGAD-ATG11 ^QQ^	AD vector with atg11 ^Q475A, Q476A^	This study
pGAD-ATG11 ^QNLNK^	AD vector with atg11 ^Q493A, N494A, L496A, N500A, K501A^	This study
pGAD-ATG11 ^TV^	AD vector with atg11 ^T511A, V512A^	This study
pGAD-ATG11 ^IA3^	AD vector with atg11 where residues 576-627 were replaced with residues 2-53 of *sc* PAI3 (aka IA3).	This study
pRS414-Cu-ATG9-PA	ATG9 tagged with Protein A, driven by the *CUP1* promoter	Meyer et al., 2022
pRS416-Cu-GFP-ATG11	GFP-ATG11 WT driven by the *CUP1* promoter	Kim et al., 2001
pRS416-Cu-GFP-ATG11ΔCC2	GFP-atg11∆ ^536-576^ driven by the *CUP1* promoter	Meyer et al., 2022
pRS416-Cu-GFP-ATG11 ^YDLL^	GFP-atg11 ^Y468A, D516A, L517A, L526A^ driven by the *CUP1* promoter	This study
pRS416-Cu-GFP-ATG11 ^YDL^	GFP-atg11 ^Y468A, D516A, L517A,^ driven by the *CUP1* promoter	This study
pRS416-Cu-GFP-ATG11 ^DLL^	GFP-atg11 ^D516A, L517A, L526A^ driven by the *CUP1* promoter	This study
pRS416-Cu-GFP-ATG11 ^IA3^	GFP-atg11 with residues 576-627 replaced with residues 2-53 of *sc* PAI3 (aka IA3). Driven by the *CUP1* promoter.	This study
**Antibodies used in this study**		
**Name**	**Identifier (Source)**	**Concentration**
“Living Colors” mouse monoclonal anti-GFP (JL-8)	Cat# 632381 (Clontech / Takara)	1:500
Peroxidase AffiniPure® Goat Anti-Mouse IgG	Cat# 115-035-003 (Jackson ImmunoResearch)	1:5000
